# Low-Abundance Proteomics Reveal Pleiotrophin and Fibroblast Growth Factor-21 as Biomarkers of Metabolic Dysfunction-Associated Steatohepatitis

**DOI:** 10.3390/ijms262210943

**Published:** 2025-11-12

**Authors:** Melissa M. Milito, Milos Mihajlovic, Alice Mallia, Stefania Ghilardi, Claudio Tiribelli, Deborah Bonazza, Natalia Rosso, Silvia Palmisano, Cristina Banfi, Pablo J. Giraudi

**Affiliations:** 1 Metabolic Liver Disease Unit, Fondazione Italiana Fegato ONLUS—Italian Liver Foundation NPO, 34149 Trieste, Italy; melissa.milito@fegato.it (M.M.M.); milos.mihajlovic@fegato.it (M.M.); ctliver@fegato.it (C.T.); natalia.rosso@fegato.it (N.R.); spalmisano@units.it (S.P.); 2 Department of Life Sciences, University of Trieste, 34127 Trieste, Italy; 3 Unit of Functional Proteomics, Metabolomics, and Network Analysis, Centro Cardiologico Monzino, IRCCS, 20138 Milan, Italy; alice.mallia@cardiologicomonzino.it (A.M.); stefania.ghilardi@cardiologicomonzino.it (S.G.); cristina.banfi@cardiologicomonzino.it (C.B.); 4 Surgical Pathology Unit, Cattinara Hospital, Azienda Sanitaria Universitaria Giuliano Isontina, 34149 Trieste, Italy; deborah.bonazza@asugi.sanita.fvg.it; 5 Surgical Clinic Division, Cattinara Hospital, Azienda Sanitaria Universitaria Giuliano Isontina, 34149 Trieste, Italy; 6 Department of Medical, Surgical and Health Sciences, University of Trieste, 34149 Trieste, Italy

**Keywords:** steatohepatitis, plasma proteomics, biomarkers

## Abstract

Metabolic dysfunction-associated steatotic liver disease (MASLD) is closely linked to comorbidities like obesity, type 2 diabetes, and cardiovascular disease. Given that liver biopsy is the diagnostic gold standard, there is a critical need for minimally invasive tests, particularly for the inflammatory form, metabolic dysfunction-associated steatohepatitis (MASH). In this discovery study, we investigated the plasma proteome to identify blood biomarkers for MASH and explored their potential tissue sources, the liver and visceral adipose tissue. Plasma low-abundance proteome profiling was performed on samples from a cohort of morbidly obese MASLD subjects (n = 90; 40 with MASH, 50 without) using Olink^®^ panels. Paired liver and visceral adipose biopsies were also analyzed. Data showed 34 significantly different plasma proteins between the two groups, including Pleiotrophin (PTN), Fibroblast growth factor-21 (FGF-21), and Hepatocyte growth factor (HGF), among others. While plasma-tissue correlation was only found for STX8, PTN and FGF-21 demonstrated the strongest associations with the histopathological features of MASH. A diagnostic model combining PTN, FGF-21, and AST achieved a robust AUC of 0.88 (95% CI: 0.84–0.97) for distinguishing MASH. Based on this discovery pilot study, circulating PTN and FGF-21 emerge as promising non-invasive biomarkers for improving patient stratification and supporting therapeutic evaluation in MASH, warranting validation in independent cohorts and future studies.

## 1. Introduction

The dramatic worldwide increase in obesity has caused an escalation of metabolic disorders, including metabolic dysfunction-associated steatotic liver disease (MASLD). With a current prevalence of over 30% worldwide, MASLD is the most common chronic liver disease and is projected to affect more than half of the population by 2040 [1,2]. Its prevalence is particularly high in obese individuals, exceeding 70% and reaching 90% in those who are morbidly obese [3]. This condition poses a significant health burden not only on the liver but also on the cardiovascular system, kidneys, and other organs, while increasing the risk of extrahepatic malignancies [4]. Recent studies have shown that the cardiometabolic burden is very high among patients with MASLD, with more than 65% of them presenting at least 3 of the cardiometabolic features, most typically obesity, pre-diabetes, and dyslipidemia [4].

The earliest and most reversible clinical presentation of MASLD includes increased intrahepatic fat accumulation (steatosis), detectable by imaging techniques such as ultrasonography. However, up to 40% of individuals with steatosis progress toward metabolic dysfunction-associated steatohepatitis (MASH), a more advanced stage marked by inflammation and liver injury. Of these individuals, about 20% may develop fibrosis and, over time, cirrhosis and, eventually, hepatocellular carcinoma [5]. Since MASLD is already recognized as a major risk factor for these severe complications, accurate and timely detection of MASH is critical for early intervention and risk minimization.

Despite being the gold standard for diagnosing MASLD, liver biopsy is unsuitable for widespread use due to its invasiveness, risk of complications, and variability in results. This has spurred the development of non-invasive alternatives. These include clinical scores like the Fatty Liver Index (FLI) and Hepatic Steatosis Index (HSI), as well as imaging techniques like ultrasound, MRI, and transient elastography [6,7,8]. In addition, various scoring systems, including the Pediatric NAFLD Fibrosis Index (PNFI), APRI, and FIB-4, are based on anthropometric measurements, liver function tests, and lipid profiles to estimate the likelihood of liver steatosis and fibrosis [9,10]. However, each of these non-invasive methods has its own limitations, such as variable sensitivity, operator dependence, or high cost, which can restrict their application in certain patient groups [11,12].

Due to the progressive nature and diagnostic limitations of MASLD, there is a clear need for novel, accurate biomarkers, particularly for MASH. These biomarkers could aid disease staging, progression assessment, and monitoring treatment response. Recent advancements in omics technologies, particularly proteomics, offer a promising avenue for discovering non-invasive, serum-based biomarkers. Affinity-based proteomics platforms, such as the Proximity Extension Assay (PEA) [13,14], have been used to identify proteins that can be integrated into non-invasive diagnostic models for various stages of MASLD [15], including steatosis [16], MASH, and fibrosis [17,18,19].

Despite holding significant potential for the non-invasive diagnosis of the MASLD spectrum, none of these biomarkers have yet been adopted into clinical practice, and only a few studies have attempted to differentiate between early stages (i.e., steatosis versus MASH). Therefore, the objective of the present study was to analyze the plasma proteome of morbidly obese subjects with and without MASH using the PEA method (Olink^®^ inflammatory, cardiometabolic, and organ damage target panels) to identify proteins that could serve as potential biomarkers for use in non-invasive algorithms to distinguish between (non-) steatotic stages and MASH.

## 2. Results

### 2.1. Morpho-Clinic Characteristics of the Subjects

The study cohort consisted of 90 morbidly obese subjects, stratified into two groups: those with MASH (n = 40) and those with No MASL/MASL (n = 50). As detailed in Table 1, the groups showed significant differences (*p* < 0.05) in several morphometric, clinical, and biochemical parameters. Although age, BMI, and the prevalence of type 2 diabetes mellitus (T2DM) were similar between groups, differences in sex distributions were observed, reflecting the higher number of women among bariatric surgery candidates and their generally milder hepatic histological profiles (86% females in No MASL/MASL vs. 60% females in MASH group, *p* = 0.007). Additionally, while 32% of the total cohort had T2DM, other major comorbidities included hypertension (24%) and obstructive sleep apnea syndrome (6.6%), as detailed in Table S4.

Notably, the MASH group had higher values for waist circumference, fasting glucose, liver transaminases (AST, ALT, and GGT), and fibrosis scores (FIB-4 and APRI). These findings are consistent with the histological characteristics of MASH.

### 2.2. Sex-Related Proteomic Differences in Plasma, Liver, and Adipose Tissue of Patients with MASLD

A proteomic analysis was conducted on 90 matched plasma, liver, and visceral adipose tissue (VAT) samples from MASLD patients using the PEA platform, targeting 276 proteins (Olink^®^ inflammation, cardiometabolic, and organ damage panels). To assess sex-related differences, the proteomic profiles of 67 female and 23 male subjects were compared.

As shown in Table 2, a total of 33 plasma proteins exhibited significant differential expressions between the sexes. In the inflammation panel, 14 proteins showed significant differences, with IL8, adenosine deaminase (ADA), and hepatocyte growth factor (HGF) being notably lower in females. Four proteins from the cardiometabolic panel, including carbonic anhydrases CA3 and CA1, as well as prolylcarboxypeptidase (PRCP), were significantly more abundant in male plasma. Additionally, 15 proteins from the organ damage panel were differentially expressed, with calcitonin-related polypeptide alpha (CALCA), natriuretic peptide C (NPPC), and carbonic anhydrase 14 (CA14) displaying markedly reduced levels in females.

In tissue samples, three proteins from the organ damage panel, aldehyde dehydrogenase 3A1 (ALDH3A1), methionine aminopeptidase 1 (METAP1), and carboxylesterase 2 (CES2), were significantly downregulated in female livers. Furthermore, four proteins, including syntaxin binding protein 3 (STXBP3), C-type lectin domain family 1 member A (CLEC1A), integrin beta 1 binding protein 1 (ITGB1BP1), and ectonucleoside triphosphate diphosphohydrolase 2 (ENTPD2), were found at higher levels in VAT samples from males.

These sex-specific proteomic variations were taken into account in all subsequent analyses investigating the MASH proteome, ensuring that the findings were not confounded by sex.

### 2.3. Plasma and Tissue Proteomics Changes in MASH

**Figure 1:**
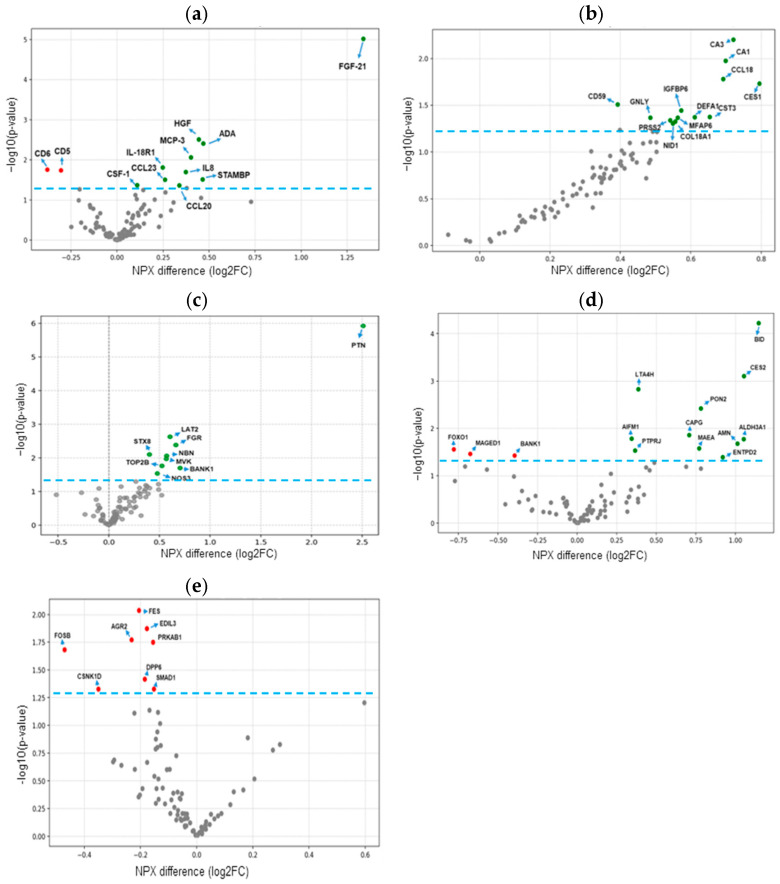
Plasma protein expression in obese individuals with and without MASH. Protein differential expressions for inflammation, cardiometabolic, and organ damage panels for plasma samples are shown in (**a**–**c**), respectively. Liver and VAT protein differential expressions for the organ damage panel in (**d**,**e**), respectively. The volcano plots show the NPX difference between MASH (n = 40) and No MASL/MASL (n = 50) subjects on the x-axis and the −log10 of the nominal *p*-value on the *y*-axis. NPX is on a log2 scale; a 1 NPX difference means a doubling of protein concentration. A dashed blue line indicates statistical significance, defined as a *p*-value less than 0.05. Green dots indicate significantly upregulated proteins, while red dots represent significantly downregulated proteins.

Following the initial analysis, we compared plasma proteome differences between the MASH and the NO MASL/MASL groups using data from the three Olink^®^ protein panels. Volcano plots (Figure 1a–c) display the results for all plasma proteins within the inflammatory, cardiometabolic, and organ damage panels, respectively. Additionally, we investigated whether the observed plasma changes in the target proteins in the organ damage panel reflect proteome alterations in the liver and VAT (Figure 1d,e).

A total of 34 circulating proteins were significantly differentially expressed in the plasma of MASH patients compared to the NO MASL/MASL group. Of these, 32 proteins showed higher expression in the MASH group, while only two proteins from the inflammation panel (CD5, T-cell surface glycoprotein CD5 and CD6, T-cell differentiation antigen CD6) showed reduced expression (Figure 1a–c, and Table S2).

**Figure 2:**
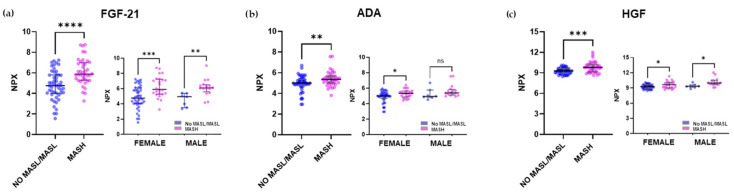
The three most differentially expressed plasma proteins in MASH subjects from the Inflammation panel, categorized by gender. Scatter plots depict the differences in NPX values for individuals with No MASL/MASL (n = 50) compared with those with MASH (n = 40). From the inflammation panel: (**a**) FGF-21 protein, (**b**) ADA protein, (**c**) HGF protein. The distribution of NPX values for both groups according to gender is also presented. Values were presented as medians with their respective 10–90 percentiles. Differences were considered statistically significant at *p*-values less than 0.05. * *p* < 0.05, ** *p* < 0.01, *** *p* < 0.001, **** *p* < 0.0001, ns—not significant.

The three most significantly increased markers in the plasma of MASH subjects from the inflammatory panel were FGF-21 (Fibroblast Growth Factor 21), HGF, and ADA (see scatter plots Figure 2a–c). For example, FGF-21 was 1.34-fold higher (*p* < 0.0001) in MASH (Median: 5.9, IQR: 5.3–7.0) than in No MASL/MASL (median: 4.76, IQR: 4.0–5.8) plasma samples.

**Figure 3:**
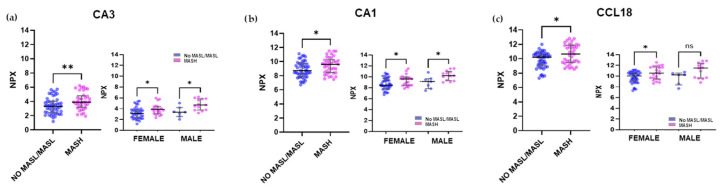
The three most differentially expressed plasma proteins in MASH subjects from the Cardiometabolic panel, categorized by gender. Scatter plots depict the differences in NPX values for individuals with No MASL/MASL (n = 50) compared with those with MASH (n = 40). From the cardiometabolic panel, (**a**) CA3 protein, (**b**) CA1 protein, (**c**) CCL18 protein. The distribution of NPX values for both groups according to gender is also presented. Values were presented as median with their respective 10–90 percentiles. Differences were considered statistically significant at *p*-values less than 0.05. * *p* < 0.05, ** *p* < 0.01, ns—not significant.

For the cardiometabolic panel, the most significant changes were observed in CA3, CA1, and CCL18 (C-C Motif Chemokine 18) plasma levels (Figure 3a–c).

**Figure 4:**
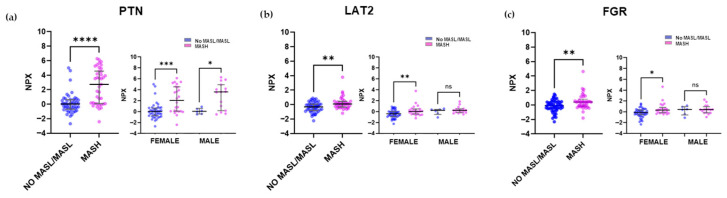
The three most differentially expressed plasma proteins in MASH subjects from the organ damage panel, categorized by gender. Scatter plots depict the differences in NPX values for individuals with No MASL/MASL (n = 50) compared with those with MASH (n = 40). From the cardiometabolic panel, (**a**) PTN protein, (**b**) LAT2 protein, (**c**) FGR protein. The distribution of NPX values for both groups according to gender is also presented. Values were presented as median with their respective 10–90 percentiles. Differences were considered statistically significant at *p*-values less than 0.05. * *p* < 0.05, ** *p* < 0.01, *** *p* < 0.001, **** *p* < 0.0001, ns—not significant.

The most significantly differentially expressed proteins from the organ damage panels were PTN (Pleiotrophin), LAT2 (Linker for activation of T cells family member 2), and FGR (FGR proto-oncogene, Src family tyrosine kinase) (Figure 4a–c). NPX changes in plasma proteins stratified by sex and MASLD status (Figure 2, Figure 3 and Figure 4) demonstrated that the observed differences remain consistent when accounting for sex, thus indicating that gender-related changes do not significantly confound this analysis.

**Figure A1:**
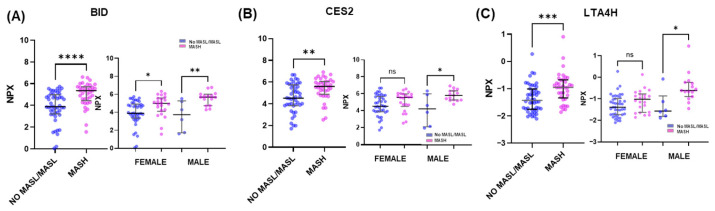
The three most differentially expressed proteins in liver specimens from the Organ damage Olink panel for MASH, categorized by gender. Scatter plots depict the differences in NPX values for individuals with No MASL/MASL (n = 50) compared with those with MASH (n = 40). (**A**) BID protein, (**B**) CES2 protein, and (**C**) LTA4H protein. The distribution of NPX values for both groups, categorized by gender, is also presented. Values were presented as medians with their respective 10–90 percentiles. Differences were considered statistically significant at *p*-values less than 0.05. * *p* < 0.05, ** *p* < 0.01, *** *p* < 0.001, **** *p* < 0.0001, ns—not significant.

**Figure A2:**
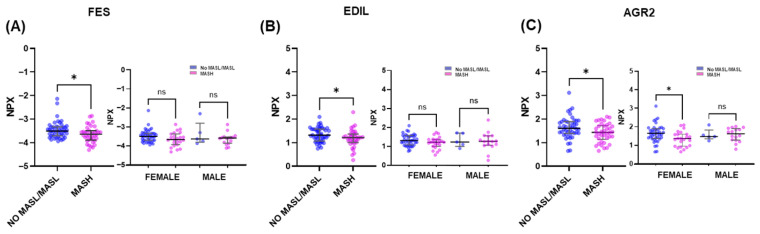
The three most differentially expressed proteins in visceral adipose specimens from the Organ damage Olink panel for MASH, categorized by gender. Scatter plots depict the differences in NPX values for individuals with No MASL/MASL (n = 50) compared with those with MASH (n = 40). (**A**) FGF21 protein, (**B**) ADA protein, (**C**) HGF protein. The distribution of NPX values for both groups according to gender is also presented. Values were presented as medians with their respective 10–90 percentiles. Differences were considered statistically significant at *p*-values less than 0.05. * *p* < 0.05, ns—not significant.

In addition to plasma findings, several markers from the organ damage panel showed significant changes in tissue samples from MASLD patients. In the liver, the three proteins out of a total of 14 showing expression changes, BID (BH3 interacting domain death agonist), LTA4H (Leukotriene A4 hydrolase), and CES2 (Carboxylesterase 2), were the most significant, with increased expression in MASH subjects (Figure 1d, Table S3). In VAT samples, from 8 proteins showing differential expression changes, FES (FES proto-oncogene, tyrosine kinase), EDIL3 (EGF-like repeats and discoidin domains 3), and AGR2 (Anterior gradient 2, protein disulphide isomerase family member) markers were among the most significantly reduced in MASH subjects (Figure 1e, Table S3). Figure A1 and Figure A2 (see Appendix A) show the scatter dot plots for the NPX differences in the cohort stratified according to MASLD and sex for the mentioned markers. Table S5 lists all the candidate markers identified by plasma PEA proteomics.

### 2.4. Correlations Analysis and Diagnostic Performance of Putative Plasma Biomarkers and MASH Clinical Parameters

**Figure 5:**
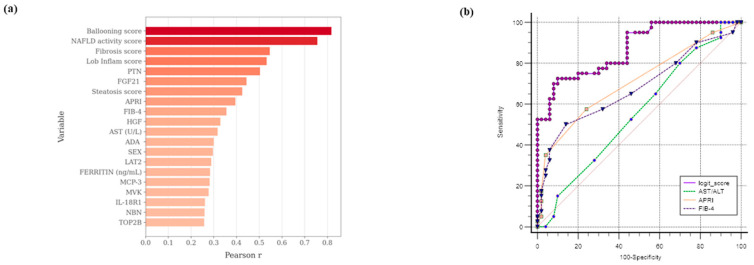
Correlation analysis and diagnostic performance of plasma proteins from the Olink^®^ panels associated with the MASH phenotype. (**a**) The horizontal bar chart displays the top 20 significant correlations with the MASH phenotype, which is histologically diagnostic. Sixty-nine parameters were included in the biserial Pearson correlation analysis to assess the association of each marker with the binary MASH status (0 = no disease, 1 = disease). Pearson’s r correlation coefficient values are shown on the x-axis for each variable on the y-axis. (**b**) ROC curves for MASH diagnosis using the logit model, which includes PTN, AST, FGF-21, and sex variables; additional ROC curves were generated for the AST/ALT ratio, APRI, and FIB-4 diagnostic clinical scores.

The next analysis focused on identifying the most significant associations between the MASH phenotype and the 34 protein markers that are differentially expressed in MASH plasma samples, along with other relevant clinical, biochemical, and histologic variables. Using point-biserial correlation, the MASH phenotype (coded as 0 for no disease and 1 for disease) was analyzed in relation to a total of 69 variables (Table S6). The top 20 significant correlations are shown in Figure 5a. As expected, the strongest associations were observed with histological parameters (including ballooning, NAFLD activity, fibrosis, and lobular inflammation scores). These were followed by plasma protein expression levels of PTN (ρ = 0.50, *p* = 0.000005) and FGF-21 (ρ = 0.44, *p* = 0.00001).

Next, the diagnostic potential of the identified plasma proteins for MASH was evaluated. Using logistic regression analysis, the optimal combination of protein markers and clinical-biochemical parameters to distinguish MASH patients accurately was determined. The model was developed through a hierarchical forward selection method and included a total of 57 numerical and two categorical variables (sex and diabetes) from 88 of the 90 subjects. The numerical variables encompassed standard clinical markers such as age, BMI, AST, ALT, GGT, fasting glucose, routine inflammation markers, and various fibrosis scores, including APRI and FIB-4. The analysis also incorporated the 34 plasma proteins previously identified as differentially expressed (see Table S7 for a full list of parameters). The statistical analysis identified four variables, PTN, AST, FGF-21, and sex, as the most predictive for MASH diagnosis, and these variables showed no relevant multicollinearity according to variance inflation factor analysis. The resulting logistic regression equation was Y = −7.6 + 0.07 × AST + 0.61 × FGF-21 + 0.47 × PTN + 1.66 × SEX. This model correctly classified 78.4% of the cohort and demonstrated strong diagnostic performance with an AUC of 0.88 (95% CI: 0.77–0.94). To assess the potential impact of overfitting and estimate model stability, an internal validation was performed using a bootstrap resampling procedure (1000 iterations), which confirmed the robustness of the model’s discriminative ability (bootstrapped AUC = 0.84, 95% CI: 0.72–0.95, see Appendix A, Figure A4, and Table S8).

Additionally, a pairwise comparison of the logit score with established MASLD scores, specifically the AST/ALT ratio (AUC: 0.55, CI: 0.45–0.66, *p* < 0.0001), APRI (AUC: 0.71, CI: 0.60–0.80, *p* = 0.00015), and FIB-4 (AUC: 0.68, CI: 0.57–0.77, *p* = 0.004), confirmed the superiority of the present model (Figure 5b).

### 2.5. Correlation Analysis Between Plasma, Liver, and VAT Proteomic Profiles

**Figure 6:**
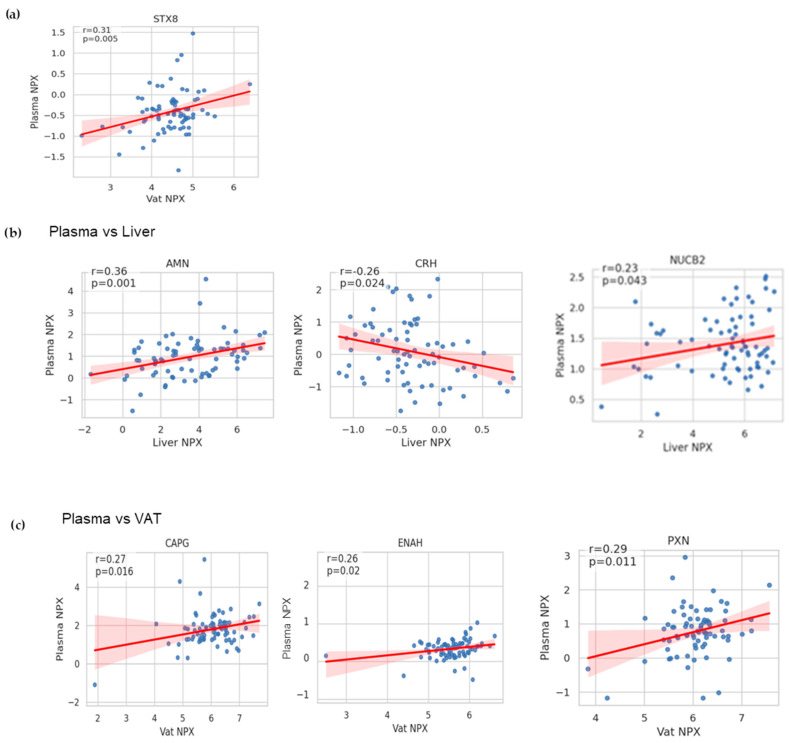
Correlation analysis of protein expression between plasma and tissue samples in MASLD. Scatter plots showing significant linear correlations between (**a**) plasma marker STX8 vs. its protein expression in VAT. Significant correlations for plasma expression levels of non-candidate diagnostic MASH markers and their expression levels in (**b**) liver or (**c**) VAT. The plot’s legend indicates Pearson’s correlation coefficient rho and the *p*-value for the association (statistical significance *p* < 0.05).

**Figure A3:**
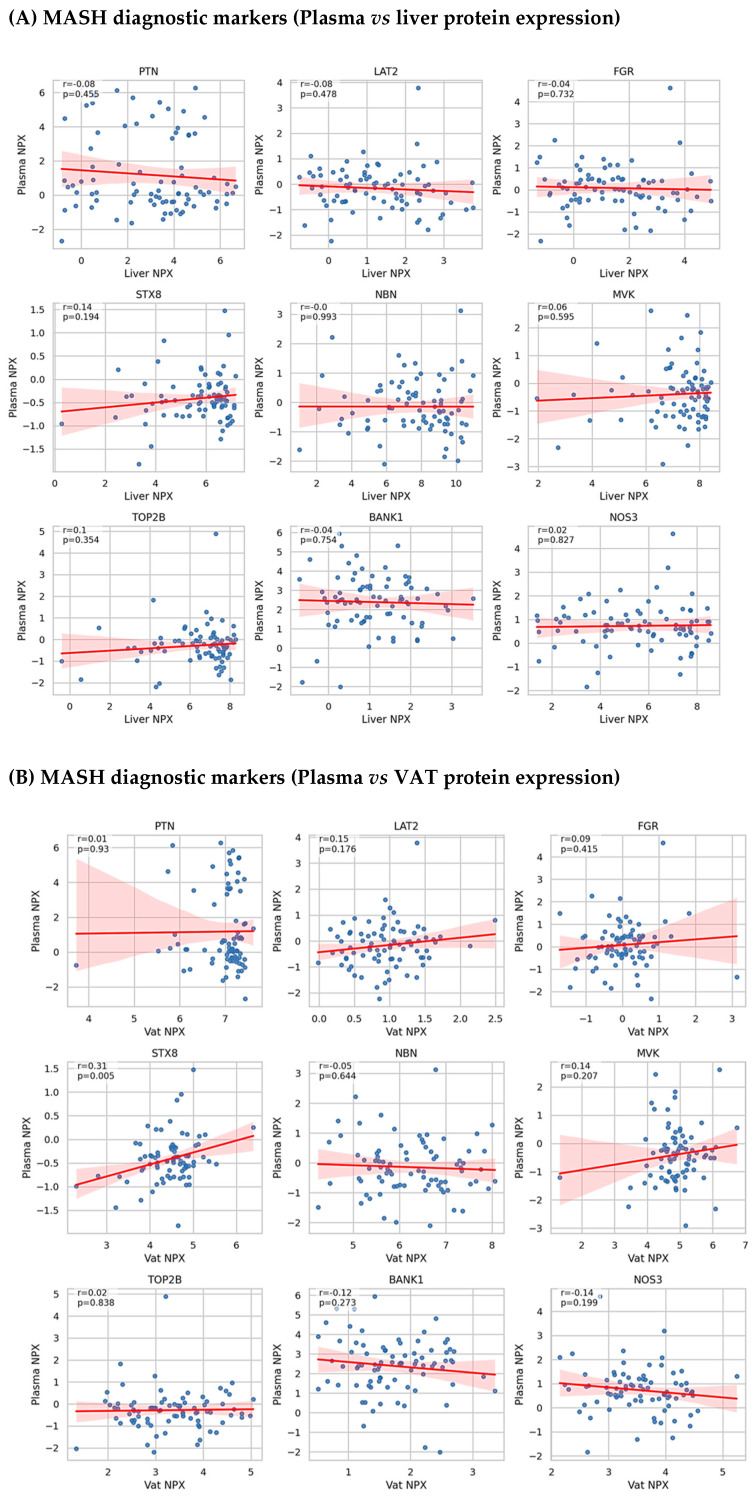
Protein expression correlations among plasma and tissue samples in MAFLD. Scatter plots showing significant linear correlations between plasma levels of MASH diagnostic candidates individuated by the organ damage panel vs. their protein expression levels in (**A**) liver and (**B**) VAT. The plot’s legend indicates Pearson’s correlation coefficient r and the *p*-value for the association.

**Figure A4:**
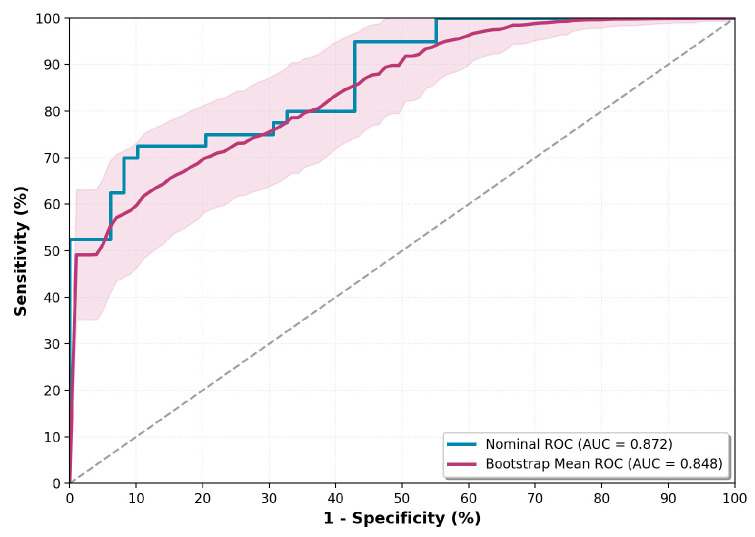
Internal validation of the logistic regression model. ROC curve showing the performance of the logistic regression model (logit score)to discriminate MASH from MASL among subjects with morbid obesity (blue line). ROC curve from the internal validation using a bootstrap resampling procedure (1000 iterations, Bordeaux line). The area under the curve (AUC) values for both analyses are indicated in the figure.

Next, it was investigated whether the plasma expression levels for the nine individual markers from the Organ Damage Olink panel were correlated with their expression values in liver and VAT samples, the two potential protein tissue sources available in the study. Pearson’s correlation analysis revealed that only the STX8 protein, which showed a change in plasma NPX of MASH subjects, exhibited a significant association between its expression values in plasma and VAT (rho = 0.31, *p* = 0.005) (Figure 6a and Figure A3).

Additionally, other significant correlations were found for proteins that lacked diagnostic capacity for MASH. Specifically, three proteins showed substantial associations between their expression in plasma and liver: AMN (ρ = 0.36, *p* = 0.001), CRH (ρ = −0.25, *p* = 0.024), and NUCB2 (ρ = 0.23, *p* = 0.043) (Figure 6b). Furthermore, three proteins were significantly correlated between plasma and VAT expression: CAPG (ρ = 0.38, *p* = 0.001), ENAH (ρ = 0.32, *p* = 0.004), and PXN (ρ = 0.29, *p* = 0.01) (Figure 6c).

## 3. Discussion

Cutting-edge proteomics technologies, particularly multiplex platforms like SomaScan and Olink^®^, have been widely used in recent years to identify plasma/serum protein patterns linked to disease. Numerous studies confirm its utility in identifying circulating biomarkers for MASLD diagnosis [17,20,21,22]. The present study explores circulating protein markers associated with MASH using three different 92-plex protein panels (Olink^®^ Inflammatory, Cardiometabolic, and Organ damage panels). The results show that 34 proteins were differentially expressed in plasma samples of morbidly obese subjects with biopsy-proven MASH. The proteins with the highest expression levels were FGF-21, HGF, and ADA in the inflammatory panel, CA3, CA1, and CCL18 in the cardiometabolic panel, and LAT2, FGR, and PTN in the organ damage panel. To the best of current knowledge, this represents the first evidence of PTN increase in the plasma of MASH subjects. The findings also emphasize the significance of sex-related differences in circulating and tissue proteomic profiles in MASLD. In the cohort used in the present study, multiple proteins showed sex-biased expression patterns, with some like ADA, HGF, and carbonic anhydrases displaying notable variation between males and females. Recent integrated proteomics and N-glycoproteomics studies using primary hepatocytes from in vivo MASLD mouse models identified over 300 sex-biased molecular signatures, including lipid metabolism-related proteins in females and inflammation or cytoskeleton-associated proteins in males [23]. Overall, these findings demonstrate that MASLD enhances sex differences at the hepatocyte and tissue proteomic level, likely affecting the plasma proteome and underscoring the importance of considering sex as a biological variable in biomarker discovery and the development of diagnostic algorithms. In this exploratory study, multiple correlation analyses were performed to obtain the top associations between these potential protein markers and MASLD histology. It was observed that PTN, FGF-21, HGF, ADA, and LAT2 were among the top 20 proteins associated with the MASH phenotype, making them promising candidates for inclusion in diagnostic models. We subsequently employed logistic regression analysis to combine these protein markers with clinic–biochemical parameters. The resulting statistical model, which includes the four best predictive variables, PTN, FGF-21, AST, and sex, exhibits the best diagnostic performance for MASH with an AUC of 0.88 (95% CI: 0.77–0.94), correctly classifying 78.4% of the cohort.

PTN is an 18 kDa cytokine that has been extensively studied for its role in the central nervous system and, more recently, for its implications in the major metabolic organs [24]. Its expression is highly upregulated in early embryonic differentiation but decreases in adulthood, remaining highest in bone and nervous tissue [25]. In peripheral organs, PTN promotes the proliferation of various cell types, including preadipocytes and pancreatic β-cells, and plays a crucial role in maintaining glucose, lipid, and whole-body insulin homeostasis [26]. Circulating PTN levels in humans have been associated with advancing age, and changes have been proposed as a potential diagnostic and prognostic tool for diseases like breast cancer, multiple sclerosis, and multiple myeloma disease status [27]. Interestingly, in a knock-out mouse model, PTN deletion was found to induce browning of periovarian adipose tissue and protect against diet-induced hepatic steatosis [28]. Additionally, PTN has been shown to be expressed in fibrotic liver, and it can be released in large amounts by activated hepatic stellate cells to aid in the early liver repair processes during fibrogenesis [29,30]. This finding aligns with the data presented in this study, showing high circulating PTN levels in the plasma of MASH patients, likely reflecting the active molecular mechanisms that sustain metabolic disturbances. Moreover, the current work also evidenced the highest PTN expression levels in visceral adipose tissue compared to liver and plasma. Unfortunately, a direct correlation between plasma levels and paired tissue biopsy levels could not be established, suggesting that additional systemic sources or complex regulatory mechanisms may be involved. This highlights that plasma protein signatures likely reflect the dynamic behavior of entire tissue programs (e.g., inflammation, metabolic alterations, extracellular matrix remodeling) from several tissues, rather than being related to simple secretion events from a single organ. To gain further biological insight, future studies should focus on integrating tissue-specific protein co-expression networks and protein interaction maps to uncover the coordinated processes underlying circulating signals. This could provide deeper insights into systems biology and support future translational applications in MASLD.

Based on the available literature, FGF-21 is a crucial hepatokine that plays a significant role in regulating lipid and glucose metabolism and exerts beneficial metabolic effects by improving lipid profiles and insulin sensitivity. It increases fatty acid oxidation and suppresses de novo lipogenesis through the activation of AMP-activated protein kinase (AMPK), sirtuin 1 (SIRT1), and peroxisome proliferator-activated receptor γ coactivator protein-1α (PGC-1α) [31,32]. Early reports have evidenced changes in FGF-21 plasma/serum levels associated with obesity [33], T2DM [34], and MASLD [35]. A systematic review by Filimidou et al. found that FGF-21 levels were significantly higher in MASLD patients than in controls, with the strongest associations observed in cases of MASH-related cirrhosis [36]. The data from the inflammatory panel support this literature and highlight the importance of including FGF-21 in future MASH diagnosis and prognosis algorithms. Regarding the adipocytokine HGF, increased plasma levels have been observed in individuals with obesity [37], metabolic syndrome (MetS) [38], and chronic liver diseases [39]. The current study extends these findings to morbidly obese subjects with MASH. Furthermore, this work provides novel evidence for an association between plasma ADA levels and MASH, as increased ADA activity had previously been described only in T2DM, acute hepatitis, and cirrhosis [40,41].

From the cardiometabolic panel, this study is also the first to report associations between MASH and changes in plasma levels of CA1 and CCL18. High serum levels of CA3 have previously been reported in MASH subjects [42] and tested as a potential marker for MASLD diagnosis [43], findings that are corroborated by our results. Lastly, the data from this study align with existing literature showing that markers like FGF-21, ADA, CES1, and CA3, which are highly expressed in the liver or adipose tissue, are among the putative plasma markers for MASH diagnosis [22,44,45]. Some of these are suggested to be involved in hepatic lipid metabolism, thus potentially contributing to MASLD. For instance, carboxylesterases (CES1, CES2) have been shown to participate in triglyceride hydrolysis and cholesterol ester hydrolysis, as well as lipogenesis and lipoprotein secretion [46]. The carbonic anhydrases are also important in lipogenesis due to the production of bicarbonate in hepatocytes, which serves as a substrate for acetyl-CoA carboxylase [47].

In addition to the current work, other multiplex platforms for low-abundance proteomics have been used to identify protein markers for MASH. Corey et al. [48] used SomaScan in bariatric patients to identify several markers (e.g., ACY1, ADAMTSL2) for diagnosing those at risk of steatohepatitis. Sanyal et al. [17] and Govaere et al. [49] also utilized SomaScan to identify different signatures associated with various histological features of the disease. Protein candidates such as ADAMTSL2, CFHR4, TREM2, and AKR1B10 have been combined in logistic diagnostic algorithms for MASH. The most recent study by Michelle L. et al. [50] included plasma levels of six proteins (including SELE, THBS2, IGFBP7, GDF15, SERPING1, and NGAMT). In relation to the development of MASH diagnostic algorithms, integrating plasma and tissue proteomics discoveries, we should mention the study of De Nardo et al. [51], who used mass spectrometry-based proteomics to analyze both plasma and liver-secreted proteins in 266 obese individuals. Their study provides a comprehensive resource of 3333 proteins, with 107 showing differential secretion in MASH, and developed a model called APASHA, which includes plasma APOF, PCSK9, AFM, S100A6, HbA1c, and AZGP1, achieving an AUROC of 0.88 for stratifying MASH. This work, alongside the present study, underscores the immense potential of novel proteomic technologies that can quantify low- and non-low-abundance proteins, integrating distinct proteomic signatures to advance knowledge of the molecular mechanisms underlying the pathology, as well as the discovery of promising biomarkers.

On the other hand, several studies have utilized the Olink^®^ discovery platform. Stiglund et al. [52] demonstrated the potential of ST1A1, ADA, Flt3L, EN-RAGE, IL-6, and IL-18 as biomarkers for MASH. Hao Wang [53] and Abozaid [22] suggested a diagnostic algorithm combining plasma FGF-21 with an additional five markers (CDCP1, FABP4, GDF15, IL-6, THBS2) or 2 (CES1 and IL18-R1) markers, respectively, in diagnostic algorithms for MASH.

The present study has several important strengths, including the use of the Olink^®^ platform, a highly sensitive affinity-based proteomics approach, and the availability of histological data for MASLD stratification. The developed diagnostic algorithm, which includes PTN, FGF-21, AST, and sex, demonstrates strong performance independent of gender, making it a valuable tool for identifying MASH in morbidly obese subjects. However, the main limitation of this pilot study is the lack of an external validation cohort. Also, the present work focused only on morbidly obese individuals, potentially limiting the applicability of the diagnostic algorithm to non-obese MASH subjects. Future studies are warranted to assess the performance of FGF-21 and PTN across the BMI spectrum, considering that both have been reported to correlate with BMI [54,55,56]. Additionally, the cross-sectional nature of the current analysis limits causal and longitudinal conclusions. Future longitudinal studies with extended follow-up are needed to assess the predictive value of these biomarkers in disease progression. In conclusion, although the present work lacks independent validation, the findings are consistent with observations from studies using the same technology, suggesting the clinical significance and potential of the assessed biomarkers.

## 4. Materials and Methods

### 4.1. Patient Enrollment and MASH Diagnosis

Ninety morbidly obese subjects enrolled in the bariatric program of the surgical department of Cattinara Hospital were included in the study. Inclusion and exclusion criteria have been previously detailed [57]. Liver and visceral adipose tissue (VAT) biopsies were obtained during laparoscopic abdominal surgery. Tissue specimens were immediately placed in RNAlater^®^ storage solution (R0901, Sigma-Aldrich, Merck, Milan, Italy) and frozen at −80 °C until further processing. Histological diagnosis of MASLD was performed according to Kleiner-Brunt criteria [58]. Liver steatosis was graded by the amount of fat in hepatocytes on hematoxylin and eosin staining. Samples with less than 5% steatosis and no injury or fibrosis were considered normal. Samples with more than 5% steatosis were classified as MASLD. The histological diagnosis of MASH and fibrosis stage was also based on the Kleiner-Brunt criteria. All subjects provided written informed consent, and all sensitive data were anonymized. The study was approved by the local Ethical Committee (Protocol N. 22979) and registered with ClinicalTrials.gov (NCT06098417).

### 4.2. Routine Blood and Proteomics Analysis

During a baseline visit two weeks before surgery, anthropometric data (age, gender, body weight, height, BMI, and waist circumference) were recorded. Fasting blood samples were collected via venipuncture using Vacuette^®^ K2E K2EDTA tubes for routine laboratory analysis. Standard biochemical parameters, including glucose, ALT, AST, GGT, total bilirubin, albumin, platelets, and lipid profiles, were measured using a Cobas 6000 analyzer (Roche Diagnostics, Monza, Italy). Diabetes was diagnosed according to ESC-EASD guidelines. The FIB-4 and APRI fibrosis scores were calculated as previously described [59].

For plasma proteomics analysis, non-fasting blood samples were collected 24 h before surgery through standard venipuncture using Vacuette K2E K2EDTA blood collection tubes (Greiner Bio-One, Kremsmünster, Austria) and maintained at 4 °C. The tubes were centrifuged at 2000× *g* for 10 min at 4 °C. Then, the resulting supernatants were centrifuged at 3000× *g* for 5 min and carefully transferred into new tubes. The supernatants were aliquoted into new tubes and promptly frozen at −80 °C to ensure long-term storage until proteome analysis.

For tissue proteomics, liver (50 mg) and VAT samples (100 mg) were homogenized in 500 μL of RIPA buffer using Bead Ruptor 4 (Omni International, Kennesaw, GA, USA) homogenizer. The homogenates were sonicated, centrifuged (at 13,000 rpm for 10 min), and the supernatants were stored at −80 °C until analysis. Total protein concentration was quantified before the proteomic assay using the bicinchoninic acid method. A predetermined amount of total protein was used for each assay well.

### 4.3. Proteome Profiling, Raw Data Generation, and Analysis

Plasma samples were analyzed using Inflammation, Cardiometabolic, and Organ damage panels on the Olink Signature Q100 (Olink Proteomics AB, Uppsala, Sweden). Liver and VAT protein homogenates were analyzed by the Olink^®^ Target 96 Organ Damage Panel. This technology employs PEA, where target proteins bind to specific oligonucleotide-labeled antibodies. Then, microfluidic real-time PCR amplification using the Olink Signature Q100 instrument is utilized to quantify the resulting DNA sequences [14]. Raw Ct data underwent quality control and were normalized considering internal and external controls with the Olink^®^ NPX Signature Software (v1.13.0). Protein levels are reported as Normalized Protein Expression (NPX) values, an arbitrary unit on a log2 scale, with higher NPX values corresponding to higher protein concentration. A list of all proteins included in each panel can be found at https://insight.olink.com/plan-study/panel-selection/target (accessed on 2 October 2025).

**Figure A5:**
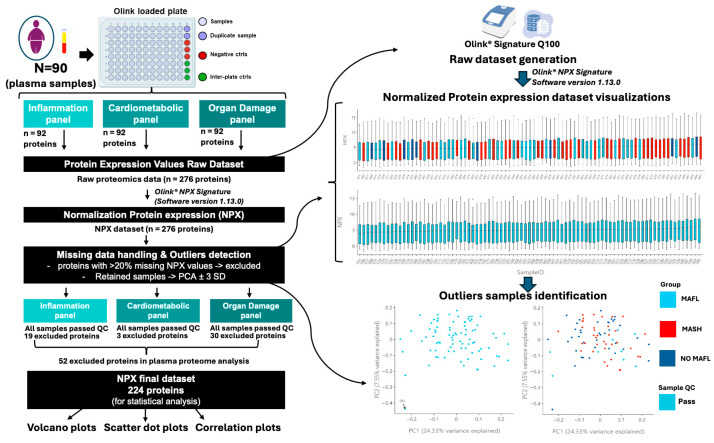
Steps for generating and analyzing proteomics datasets. All samples were analyzed in a single batch under identical conditions using Olink^®^ Target panels. Raw cycle threshold (Ct) data were processed with Olink’s standard pipeline to obtain Normalized Protein eXpression (NPX) values, which are already adjusted using internal, negative (NEG), and inter-plate (IPC) controls and are expressed on a log_2_ scale. Each plate included three IPC and three NEG controls to monitor assay performance and determine the detection threshold. One biological sample was run in duplicate across plates to assess precision and reproducibility. All samples passed Olink’s internal quality control checks and were retained for downstream analysis. Proteins with >20% missing NPX values were excluded, and outliers were identified via PCA and NPX distribution inspection. For plasma samples, a total of 224 proteins were included in the final analysis (excluding 19, 3, and 30 proteins from inflammation, cardiometabolic, and organ damage, respectively). The same steps were followed in generating the raw datasets from liver and VAT homogenate samples, which were run for the organ damage panel, resulting in the exclusion of 30 and 14 proteins, respectively, for each tissue sample batch.

Data visualization, exploration, and initial statistical analysis were performed using the Olink web-based analysis platform (https://olink.com/software/olink-analyze accessed on 2 October 2025). The NPX datasets were uploaded into the application, and quality controls were checked and passed for all assessed samples. Consistent with prior PEA/antibody-based proteomics literature, we excluded proteins missing in >20% of samples to maintain robustness of downstream analysis [60]. For plasma samples, 52 proteins with a missing frequency greater than 20% were excluded from the final analysis (19 from inflammation, 3 from cardiometabolic, and 30 from the organ damage panel, leaving 224 plasma proteins for downstream analysis. Similarly, 30 and 14 proteins with higher missing rates were excluded from the liver and VAT analyses. Reported *p*-values from *t*-tests were adjusted for multiple comparisons using the Benjamini–Hochberg method, with a threshold to determine statistical significance in adjusted *p*-value less than 0.05 (see Tables S2 and S3). In scatter dot plots, results are presented as NPX median values and inter-quartile range (IQR) (see further details in Supplementary Materials: Methods, Tables S1–S3 and S9, and Appendix A Figure A5).

### 4.4. Statistical Methods

The samples were categorized based on the histological diagnosis of the full spectrum of MASLD (No MASL, MASL, and MASH). Statistical comparisons among MASH and No MASL/MASL groups were performed on the NPX datasets. Continuous variables were expressed as mean ± standard deviation (SD) or median (and interquartile range, IQR), and categorical variables as numbers or percentages. Chi-square tests with appropriate corrections were used for categorical variables. Normally distributed continuous variables were analyzed with independent *t*-tests and ANOVA. Non-parametric tests (Mann–Whitney and Kruskal–Wallis with post hoc analysis) were applied to variables that failed the D’Agostino & Pearson omnibus normality test.

Pearson’s method was used for correlation analysis between plasma markers’ NPX values and clinical–histological parameters. Correlation statistical analysis was performed using GraphPad Prism 10.2.0 and Julius AI (https://julius.ai/; accessed on 29 August 2025). Moreover, Julius AI was used for generating correlation, top 20 associations plots, and volcano plots. Scripts to obtain all plots and associated statistics are included in the Supplementary Materials.

Logistic regression and internal validation. Logistic regression analyses were conducted using NCSS 11 Software (NCSS, LLC, Kaysville, UT, USA). The model was built with a hierarchical forward selection method, utilizing a switching one-way logistic subset selection process to identify the best combination of clinical, biochemical, and protein variables that distinguished the MASH phenotype from No-MAFLD/MAFLD. The dataset included 59 predictors (57 continuous and two categorical: sex and diabetes). The model was allowed up to 100 iterations, with a final degree of freedom of 5. Multicollinearity among predictors was evaluated with variance inflation factors (VIFs) and condition indices; values over 5 and 30, respectively, indicated collinearity. In such cases, redundant predictors were removed or combined based on biological relevance. The final model (including PTN, FGF-21, AST, and sex) was re-implemented in Python for internal validation using bootstrap resampling (1000 iterations). Model performance was reported as the mean and 95% confidence interval (CI) for AUC, sensitivity, and specificity. ROC curves and statistical comparisons with established non-invasive indices (AST/ALT, APRI, FIB-4) were performed using DeLong’s statistical test.

## 5. Conclusions

Our study found that plasma PTN and FGF-21 levels are significantly linked to MASH in morbidly obese individuals. While many diagnostic algorithms exist, none have been widely adopted. We propose that a promising path forward involves validating the most robust biomarkers identified in recent studies, including two of the reporters herein, PTN and FGF-21, and integrating them into new diagnostic algorithms.

## Figures and Tables

**Table 1:** Morphometrics and clinic–biochemical characteristics of MASLD groups.

Variable	NO MASL/MASL (n = 50)	MASH (n = 40)	*p* Value
AGE (years)	43 ± 8.5	46 ± 11	0.32
Gender female (n, %)	43, 86%	24, 60%	0.007
BMI (kg/m^2^)	42 ± 5	44 ± 5	0.26
WAIST CIRCUMFERENCE (cm)	126 ± 14	134 ± 14	0.028
FASTING GLUCOSE (mg/dL)	99 (92.0–111.0)	107 (97.0–124.0)	0.032
DIABETES (n, %)	16, 32%	13, 32%	0.82
AST (UI)	21 (16.7–25.2)	25 (20.0–33.0)	0.0039
ALT (UI)	24 (16.0–32.0)	31 (21.0–40.7)	0.013
GGT (UI)	24 (18.0–30.2)	32.5 (24.0–60.0)	0.0009
TRYGLICERIDES (mg/dL)	121 (102.0–167.8)	140 (114–211)	0.15
TOT COL (mg/dL)	195 (164–226)	204 (173–239)	0.2321
HDL COL (mg/dL)	48 (41.7–55.2)	45 (39.2–50.7)	0.085
INSULIN (mIU/L)	16 (11.6–22.7)	18 (12.6–28.3)	0.30
HbA1c (%)	5.8 (5.4–6.2)	6 (5.6–6.5)	0.083
FIB-4	0.72 (0.54–0.89)	0.87 (0.68–1.36)	0.0037
APRI	0.19 (0.15–0.24)	0.27 (0.21–0.41)	<0.0001
STEATOSIS 0/1/2/3	10/30/9/1	0/14/14/12	<0.0001
BALOONING 0/1/2	50/0/0	0/24/16	<0.0001
LOBULAR INFLAMMATION 0/1/2	28/21/0	0/34/6	<0.0001
FIBROSIS 0/1/2	27/11/12	0/0/40	<0.0001

BMI, body mass index; AST, aspartate aminotransferase; ALT, alanine aminotransferase; GGT, gamma-glutamyl transferase; HbA1c, glycated hemoglobin; HDL, high-density lipoprotein; LDL, low-density lipoprotein; *p* < 0.05 was considered statistically significant. Data are presented as mean ± SD or median with (IQR) for normally and non-normally distributed continuous variables, respectively, and as number (%) for categorical variables. The *t*-test was used to identify significant differences in normally distributed continuous variables, while the Mann–Whitney and Kruskal–Wallis tests were used for non-normally distributed variables. The Chi-square test was employed for categorical variables.

**Table 2:** Differential protein expression in plasma, liver, and visceral adipose tissue samples between female and male MASLD patients.

Olink Panel	Sample Type	Protein	Uniprot ID	NPX Difference (log2)	*p* Value
Inflammation	Plasma	IL8	P10145	−0.64	0.001
		ADA	P00813	−0.53	0.008
		HGF	P14210	−0.48	0.011
		CST5	P28325	−0.26	0.017
		TNFB	P01374	0.25	0.018
		AXIN1	O15169	−0.52	0.018
		IL-18R1	Q13478	−0.26	0.024
		CCL11	P51671	−0.28	0.026
		SLAMF1	Q13291	−0.31	0.026
		GDNF	P39905	−0.22	0.032
		4E-BP1	Q13541	−0.86	0.037
		STAMBP	O95630	−0.53	0.038
		SIRT2	Q8IXJ6	−0.62	0.041
		CCL4	P13236	−0.29	0.045
Cardiometabolic	Plasma	CA3	P07451	0.94	0.005
		CA1	P00915	0.88	0.005
		PRCP	P42785	0.54	0.031
		IGFBP6	P24592	0.70	0.04
Organ damage	Plasma	CALCA	P01258	−1.172	0.000
		NPPC	P23582	−1.084	0.005
		CA14	Q9ULX7	−0.2876	0.007
		PGF	P49763	−0.2334	0.008
		BANK1	Q8NDB2	−0.9622	0.009
		ADGRG1	Q9Y653	−0.7946	0.014
		NOS3	P29474	−0.5459	0.016
		PVALB	P20472	−0.7871	0.019
		TNNI3	P19429	−1.317	0.022
		MVK	Q03426	−0.5949	0.025
		PLIN1	O60240	0.3254	0.037
		YES1	P07947	−0.5204	0.038
		LAT2	Q9GZY6	−0.3985	0.039
		INPPL1	O15357	−0.6139	0.044
		LHB	P01229	0.5065	0.047
	Liver	ALDH3A1	P30838	−1.246	0.011
		METAP1	P53582	−0.3337	0.011
		CES2	O00748	−0.7181	0.046
	VAT	STXBP3	O00186	0.1936	0.008
		CLEC1A	Q8NC01	0.2559	0.023
		ITGB1BP1	O14713	0.217	0.04
		ENTPD2	Q9Y5L3	0.4229	0.049

The NPX difference represents the log2 fold change, with positive values indicating higher protein abundance in males. The corresponding linear fold change is calculated as 2 to the power of the NPX difference in the Fold Change column. Statistical significance (*p*-value) was determined using an independent samples *t*-test, with a significance threshold of *p* < 0.05. VAT, visceral adipose tissue.

## Data Availability

The original contributions presented in the study are included in the article/Supplementary Materials. For further inquiries about raw low-abundance proteomics datasets, do not hesitate to get in touch with the corresponding author.

## Supplementary Materials

The supporting information can be downloaded at: https://www.mdpi.com/article/10.3390/ijms262210943/s1.

## Abbreviations

The following abbreviations are used in this manuscript:

ADA	Adenosine Deaminase
ALDH3A	Aldehyde Dehydrogenase 3A1
AMN	Amnionless
APRI	Aspartate aminotransferase to platelet ratio index
BID	BH3 interacting domain death agonist
CA1	Carbonic anhydrase 1
CA3	Cabonic anhydrase 3
CA14	Carbonic anhydrase 14
CALCA	Calcitonin-related polypeptide alpha
CCL18	C-C Motif Chemokine Ligand 18
CD5	T-Cell Surface Glycoprotein CD5
CD6	T-Cell Differentiation Antigen CD6
CES1	Carboxylesterase 1
CES2	Carboxylesterase 2
CLEC1A	C-type lectin domain family 1 member A
CRH	Corticotropin-releasing hormone
EDIL3	EGF-like repeats and discoidin domains 3
ENAH	Enah/vasp-like
ENTPD2	Ectonucleoside triphosphate diphosphohydrolase 2
FES	FES proto-oncogene, tyrosine kinase
FGF-21	Fibroblast Growth Factor 21
FIB-4	Fibrosis-4 Index
GGT	Gamma-glutamyl transferase
HGF	Hepatocyte Growth Factor
HSI	Hepatic Steatosis Index
IL-8	IL-8, Interleukin-8
IQR	Interquartile range
ITGB1BP1	Integrin beta 1 binding protein 1
LAT2	Linker for activation of T cells family member 2
LT4H	Leukotriene A4 hydrolase
METAP1	Methionine Aminopeptidase 1
MetS	Metabolic Syndrome
NPPC	Natriuretic Peptice C
NPX	Normalized Protein Expression
Nucleobindin	Nucleobindin 2
PEA	Proximity Extension Assay
PNFI	Pediatric NAFLD Fibrosis Index
PRCO	Prolylcarboxypeptidase
PTN	Pleiotrophin
ROC	Receiver Operating Characteristic
STAMBP	STAM Binding Protein
TG	Triglycerides
T-Chol	Total Cholesterol
T2DM	Type 2 Diabetes Mellitus
VAT	Visceral Adipose Tissue
STX8	Syntaxin-8
CAPG	Macrophage-capping protein
PXN	Paxillin
NUCB2	Nucleobindin-2
