# Supplementary material for: Low-Abundance Proteomics Reveal Pleiotrophin and Fibroblast Growth Factor-21 as Biomarkers of Metabolic Dysfunction-Associated Steatohepatitis
Source: Int J Mol Sci. 2025 Nov 12;26(22):10943. doi: 10.3390/ijms262210943 (PMC12652421; doi:10.3390/ijms262210943)
Supplement: Supplementary file 1 [file ijms-26-10943-s001.zip › 2025.11.10_Supplementary Information_MASH.pdf]

# Supplementary Materials

**Low-Abundance Proteomics Reveal Pleiotrophin and Fibroblast Growth Factor-21 as Biomarkers of Metabolic Dysfunction-Associated Steatohepatitis**

Melissa M. Milito [1 2 (†)], Milos Mihajlovic [1(†)], Alice Mallia [3], Stefania Ghilardi [3], Claudio Tiribelli [1], Deborah Bonazza [4], Natalia Rosso [1], Silvia Palmisano [1, 5, 6], Cristina Banfi [3], and Pablo J. Giraudi [1]

[1] Fondazione Italiana Fegato ONLUS – Italian Liver Foundation NPO, Metabolic Liver Disease Unit, Trieste, Italy

[2] Department of Life Sciences, University of Trieste, UNITS, Trieste, Italy

[3] Unit of Functional Proteomics, Metabolomics, and Network analysis, Centro Cardiologico Monzino, IRCCS, Milan, Italy

[4] Surgical Pathology Unit, Cattinara Hospital, ASUGI, 34149 Trieste, Italy

[5] Surgical Clinic Division, Cattinara Hospital, ASUGI, 34149 Trieste, Italy

[6] Department of Medical, Surgical and Health Sciences, University of Trieste, 34149 Trieste, Italy

(†) These authors contributed equally to this work and shared the first authorship

*** Correspondence:** Pablo J. Giraudi, pablo.giraudi@fegato.it

Page 2. **Index**

Page 3 **Methods: Proteome profiling and analysis**

Page 4-6. **Logistic regression model report**

Page 7-10. **Python scripts**

**Supplementary tables (xlsx spreadsheets):**

**Table S1.** Excluded proteins from the statistical analysis for the three panels

**Table S2.** Volcano plots statistical data for Olink Inflammation, Cardiometabolic, and Organ damage proteomic panels when comparing MASH vs. No MASL/MASL plasma samples

**Table S3.** Volcano plots statistical data for Olink Inflammation, Cardiometabolic, and Organ damage proteomic panels when comparing MASH vs. No MASL/MASL in liver and VAT samples

**Table S4.** Comorbidities present in the morbidly obese cohort

**Table S5.** Selected candidates according to statistics from the plasma proteomics panels for MASH

**Table S6.** Variables included in the point-biserial correlations analysis

**Table S7.** List of all the parameters (n=59) included in the logistic regression model analysis

**Table S8.** ROC's performance metrics of logit score, and for internal bootstrapping statistical validation

**Table S9.** NPX values for a plasma sample run in duplicate

***Methods***

**Proteome profiling data generation and analysis**

Data generation and normalization pipeline: All samples were analyzed in a single batch under identical experimental conditions for each panel. Raw outputs from the instrument (cycle threshold, Ct values) for each panel were processed with Olink's software to generate NPX values. These NPX values are obtained after Olink's normalization pipeline, which includes within-run normalization using internal and negative (NEG) controls, and inter-run normalization using inter-plate controls (IPCs). Thus, while the raw Ct data are non-normalized, the NPX values we report are already normalized and expressed on a $log_2$ scale, as provided by the Olink platform; no additional transformation was applied. Each plate contained three IPC and NEG controls to correct for technical variation and to define the no-signal threshold. One biological sample was also run in duplicate across plates to evaluate assay precision and reproducibility. Inter-run and intra-assay coefficients of variation (CVs) for the inflammation panel are reported in Table S8. The mean intra-assay CVs were below 10%, consistent with Olink's performance specifications, while CVs above 15% were observed only for proteins with very low NPX values (mean = 0.30, IQR = 0.21–0.70).

Missingness and outlier handling: Proteins with > 20% missing NPX values were excluded from downstream analyses, as extensive missingness in PEA assays typically reflects concentrations below the limit of detection (LOD) or assay-specific technical variation. This 20% threshold is widely adopted in Olink-based studies to ensure statistical robustness. Outlier detection was conducted through PCA and visual inspection of NPX distributions; samples or features exceeding ± 3 SD from the mean of the first two principal components were removed.

All samples successfully passed Olink's internal quality control checks and were included in the downstream analyses. A flow-chart summarizing the steps for ptrotemics data generation is presented as supplementary figure, see Figure A5.

## *Logistic regression models report*

### Model for Logit(MASH diagnosis) = XB when diagnosis_outcome = 1

7.57695248874183 + 0.066647649512763*AST__U_L_ + 0.614305598172392*FGF21 + 0.465190705138323*PTN + 1.66004937491936*SEX

**Run Summary**

| Item | Value | Item | Value |
|---|---|---|---|
| Y Variable | diagnosis_outcome | Rows Processed | 90 |
| Reference Value | 0 | Rows Used | 88 |
| Number of Y-Values | 2 | Rows for Validation | 0 |
| Frequency Variable | None | Rows X's Missing | 2 |
| Numeric X Variables | 57 | Rows Freq Miss. or 0 | 0 |
| Categorical X Variables | 2 | Rows Prediction Only | 0 |
| Final Log Likelihood | -37.35680 | Unique Rows (Y and X's) | 88 |
| Model $R^2$ | 0.38388 | Sum of Frequencies | 88 |
| Actual Convergence | 5.563351E-09 | Likelihood Iterations | 6 |
| Target Convergence | 1E-06 | Maximum Iterations | 100 |
| Model D.F. | 5 | Completion Status | Normal Completion |
| Priors | Equal | | |
| Subset Selection Method | Hierarchical Forward Selection with Switching | | |

**Y Variable Summary**

| Y diagnosis_outcome | Count | Unique Rows (Y and X's) | Y Proportion | Y Prior | $R^2$ (Y vs Pred. Probability) | Percent Correctly Classified |
|---|---|---|---|---|---|---|
| 0 | 48 | 48 | 0.54545 | 0.50000 | 0.42022 | 83.333 |
| 1 | 40 | 40 | 0.45455 | 0.50000 | 0.42022 | 72.500 |
| Total | 88 | 88 | | | | 78.409 |

**Subset Selection Summary**

Subset Selection Method = Hierarchical Forward Selection with Switching

| No. Terms | No. X's | Log Likelihood | $R^2$ Value | $R^2$ Change |
|---|---|---|---|---|
| 1 | 1 | -60.63281 | 0.00000 | 0.00000 |

| | | | | |
|---|---|---|---|---|
| 2 | 2 | -48.31820 | 0.20310 | 0.20310 |
| 3 | 3 | -42.51728 | 0.29877 | 0.09567 |
| 4 | 4 | -40.05146 | 0.33944 | 0.04067 |
| 5 | 5 | -37.35680 | 0.38388 | 0.04444 |

**Subset Selection Detail**

Subset Selection Method = Hierarchical Forward Selection with Switching

| Step | Action | No. of Terms | No. of X's | Log Likelihood | Term Entered | Term Removed |
|---|---|---|---|---|---|---|
| 1 | Add | 1 | 1 | -60.63281 | Intercept | |
| 2 | Add | 2 | 2 | -48.31820 | PTN | |
| 3 | Add | 3 | 3 | -42.51728 | AST__U_L_ | |
| 4 | Add | 4 | 4 | -40.05146 | FGF21 | |
| 5 | Add | 5 | 5 | -37.35680 | SEX | |

**Classification Table**

| | Estimated | | |
|---|---|---|---|
| **Actual** | **0** | **1** | **Total** |
| **0** | 40 | 8 | 48 |
| **1** | 11 | 29 | 40 |
| **Total** | 51 | 37 | 88 |

Percent Correctly classified = 78.4%

**Variables, Model Tab**

-- Variables ------------------------------------------------------------------------------------------------------------------------------

| | |
|---|---|
| Y: | diagnosis_outcome |
| Reference Value: | 0 |
| Numeric X's: | Age, BMI__kg_m2_, AST__U_L_, ALT__U_L_, GGT__U_L_, Alkaline_Phosphatase__U_L_, Fasting_Glucose__mg_dL_, Triglycerides__mg_dL_, HDL_cholesterol__mg_dL_, PLATELETS__x103_ÂµL_, AST_ALT, APRI, FIB_4, FORNS, Lymphocytes__x103__µL_, Neutrophils__x103__µL_, Platelets_Lymphocytes, Neutrophils_Lymphocytes, PNI, Monocytes___x103__µL_, Lymphocytes_Monocytes, SII, FGF21, HGF, ADA, MCP_3, IL_18R1, CD6, CD5, IL_8, STAMBP, CCL23, CSF_1, CCL20, CA3, CA1, CCL18, CES1, CD59, CST3, DEFA1, MFAP5, IGFBP6, GNLY, PRSS2, COL18A1, |
| NID1, | |
| | PTN, LAT2, FGR, STX8, NBN, MVK, TOP2B, BANK1, NOS3 |
| Categorical X's: | SEX, diabetes |
| Default Recoding Scheme: | Compare Each with Reference Value |
| Default Reference Value: | First after Sorting |
| Frequencies: | <Empty> |
| Validation Filter: | <Empty> |

-- Regression Model --------------------------------------------------------------------------------------------------------------
-------

| | |
|---|---|
| Terms: | 1-Way |
| Remove Intercept | Unchecked |

**Code scripts from Julius AI used to generate volcano and correlation plots in Python software.**

***# Generation of volcano plots using Python 3.12***

```
Volcano plots, set-ups and others
pip --version
pip install numpy pandas matplotlib

import os
import sys
import pandas as pd

# Set up the path to the folder containing the data files
folder_path = 'C:\\path\\to\\your\\folder'

# Add the folder to the Python path
sys.path.append(folder_path)

# Function to find and read CSV files in the folder
def read_csv_files(folder_path):
    csv_files = [f for f in os.listdir(folder_path) if f.endswith('.csv')]
    data_frames = [pd.read_csv(os.path.join(folder_path, file)) for file in 
csv_files]
    return data_frames

# Read CSV files
data_frames = read_csv_files(folder_path)

# Perform your data analysis (example: concatenating all data frames)
combined_data = pd.concat(data_frames, ignore_index=True)

# Save the analysis result in the same folder
output_file = os.path.join(folder_path, 'analysis_result.csv')
combined_data.to_csv(output_file, index=False)

print(f"Analysis results saved to {output_file}")

# Save the script as data_analysis.py.
# Run the Script
# Open Command Prompt.
# Navigate to the directory where your script is saved:
cd C:\path\to\your\script
```

```
# Run the script using Python:
python data_analysis.py


# Volcano plot code
import pandas as pd
import numpy as np
import matplotlib.pyplot as plt

# Load the data
FILEPATH = 'Inflammation_data_volcano.xlsx'
df = pd.read_excel(FILEPATH, sheet_name='Foglio1')

# Calculate the negative log10 of the p-values
df['neg_log10_pvalue'] = -np.log10(df['p.value'])

# Create a mask for significant p-values
significant_mask = df['p.value'] < 0.05

# Plotting
plt.figure(figsize=(10, 6))
plt.scatter(df['estimate'][~significant_mask],
df['neg_log10_pvalue'][~significant_mask], color='gray', label='Not Significant')
plt.scatter(df['estimate'][significant_mask],
df['neg_log10_pvalue'][significant_mask], color='red', label='Significant (p <
0.05)')
plt.title('Volcano Plot of Differential Protein Expression')
plt.xlabel('Estimate')
plt.ylabel('-Log10(p-value)')
plt.legend()
plt.grid(True)
plt.show()
print('Volcano plot generated successfully.')


# Adjusting the plot with new color conditions
plt.figure(figsize=(10, 6))
# Non-significant values
plt.scatter(df['estimate'][~significant_mask],
df['neg_log10_pvalue'][~significant_mask], color='gray', label='Not Significant')
# Significant values with Estimate > 0
plt.scatter(df['estimate'][(significant_mask) & (df['estimate'] > 0)],
df['neg_log10_pvalue'][(significant_mask) & (df['estimate'] > 0)], color='green',
label='Significant Positive (p < 0.05)')
```

```
# Significant values with Estimate < 0
plt.scatter(df['estimate'][(significant_mask) & (df['estimate'] < 0)],
df['neg_log10_pvalue'][(significant_mask) & (df['estimate'] < 0)], color='red',
label='Significant Negative (p < 0.05)')
plt.title('Volcano Plot of Differential Protein Expression')
plt.xlabel('Estimate')
plt.ylabel('-Log10(p-value)')
plt.legend()
plt.grid(True)
plt.show()
print('Updated volcano plot generated successfully.')

# Plotting without the legend
plt.figure(figsize=(10, 6))
plt.scatter(df['estimate'][~significant_mask],
df['neg_log10_pvalue'][~significant_mask], color='gray')
plt.scatter(df['estimate'][(significant_mask) & (df['estimate'] > 0)],
df['neg_log10_pvalue'][(significant_mask) & (df['estimate'] > 0)], color='green')
plt.scatter(df['estimate'][(significant_mask) & (df['estimate'] < 0)],
df['neg_log10_pvalue'][(significant_mask) & (df['estimate'] < 0)], color='red')
plt.title('Volcano Plot of Differential Protein Expression')
plt.xlabel('Estimate')
plt.ylabel('-Log10(p-value)')
plt.grid(True, linestyle='--', linewidth=0.5)
plt.show()
print('Volcano plot without legend generated successfully.')
```

***# Generation of bar plot for top 15 correlations with NASH phenotype using Python 3.12***

```
import pandas as pd
import numpy as np
from scipy.stats import pearsonr
import matplotlib.pyplot as plt
import seaborn as sns

# Load data
file = 'Corr_histo&OlinkNASH_34_markers.xlsx'
df = pd.read_excel(file, sheet_name='data_histo&olink', engine='calamine')

df_num = df.apply(pd.to_numeric, errors='coerce')
df_num = df_num.loc[:, ~df_num.columns.duplicated()]
```

```
target = 'NASH disease'

# Compute correlations and p-values
corrs = {}
pvals = {}
for col in df_num.columns:
    if col == target:
        continue
    valid = df_num[[target, col]].dropna()
    if valid.shape[0] >= 3:
        r, p = pearsonr(valid[target], valid[col])
    else:
        r, p = np.nan, np.nan
    corrs[col] = r
    pvals[col] = p

# Build DataFrame
df_rel = pd.DataFrame({'variable': pd.Series(corrs).index, 'r':
pd.Series(corrs).values, 'p_value': pd.Series(pvals).values})
# Filter significant
df_sig = df_rel[(df_rel['p_value'] < 0.05) & df_rel['r'].notna()]
# Top 15 by |r|
df_top15 =
df_sig.reindex(df_sig['r'].abs().sort_values(ascending=False).index).head(15)

# Print table
print(df_top15.reset_index(drop=True))

# Plot
plt.rcParams['font.family'] = 'serif'
cmap = plt.get_cmap('coolwarm')
max_abs = df_top15['r'].abs().max()
norm = plt.Normalize(-max_abs, max_abs)
colors = [cmap(norm(val)) for val in df_top15['r'][::-1]]

fig, ax = plt.subplots(figsize=(9, 6))
plt.subplots_adjust(left=0.25, right=0.85, top=0.85, bottom=0.15)
ax.set_axisbelow(True)
ax.barh(df_top15['variable'][::-1], df_top15['r'][::-1], color=colors)
ax.set_title('Top 15 Significant Correlations with NASH disease', pad=15,
fontsize=16, fontweight='semibold', color='#222222')
ax.set_xlabel('Pearson r', labelpad=10, fontsize=14, color='#333333')
ax.set_ylabel('Variable', labelpad=10, fontsize=14, color='#333333')
ax.tick_params(axis='y', labelsize=12, colors='#555555')
ax.tick_params(axis='x', labelsize=12, colors='#555555')
```

```
ax.xaxis.grid(True, color='#E0E0E0')
for spine in ax.spines.values():
    spine.set_color('#333333')
    spine.set_linewidth(0.5)
plt.show()
```

***# Generation of scatterplots and correlation plots using Python 3.12***

```
# Plot scatterplots for CAPG, ENAH, PXN, STX8 for Plasma vs Liver and Plasma vs
VAT
import pandas as pd
import numpy as np
import matplotlib.pyplot as plt
import seaborn as sns
from scipy.stats import pearsonr

# Load data
df_pl = pd.read_excel('data_plasma_final.xlsx', engine='openpyxl')
df_li = pd.read_excel('data_liver_final.xlsx', engine='openpyxl')
df_va = pd.read_excel('data_vat_final.xlsx', engine='openpyxl')
for df in (df_pl, df_li, df_va): df.columns = [c.strip() for c in df.columns]
# Proteins of interest
proteins = ['CAPG','ENAH','PXN','STX8']
# Merge dataframes on SampleID
df = df_pl[['SampleID'] + proteins].merge(
    df_li[['SampleID'] + proteins], on='SampleID', suffixes=('_plasma','_liver')
).merge(
    df_va[['SampleID'] + proteins], on='SampleID'
)
# Rename VAT columns
df = df.rename(columns={p: p + '_vat' for p in proteins})
```

```
# Clean infinities and drop missing
df = df.replace([np.inf, -np.inf], np.nan).dropna()

# Plot function
def plot_proteins(prots, tissue):
    fig, axes = plt.subplots(1, len(prots), figsize=(4*len(prots), 4),
sharey=True)
    fig.suptitle(f'Plasma vs {tissue.capitalize()}', fontsize=14)
    for ax, prot in zip(axes, prots):
        x = df[prot + f'_{tissue}']
        y = df[prot + '_plasma']
        sns.regplot(x=x, y=y, ax=ax, scatter_kws={'s':20},
line_kws={'color':'red'}, ci=95)
        r, p = pearsonr(x, y)
        ax.set_title(prot)
        ax.text(0.05, 0.85, 'r=' + str(round(r,2)) + '\
p=' + str(round(p,3)), transform=ax.transAxes,
                bbox=dict(boxstyle='round', facecolor='white', alpha=0.6))
        ax.set_xlabel(tissue.capitalize() + ' NPX')
        if ax is axes[0]: ax.set_ylabel('Plasma NPX')
    plt.tight_layout(rect=[0,0,1,0.95])
    plt.show()

sns.set(style='whitegrid')
plot_proteins(proteins, 'liver')
plot_proteins(proteins, 'vat')
```

***# Testing multicollinearity for variables in logit model using Python 3.12***

```
# Step 1: Compute VIF for predictors in the NCSS logistic model
import pandas as pd
from statsmodels.stats.outliers_influence import variance_inflation_factor
import numpy as np

# Prepare design matrix with intercept and predictors
X = df_clean[['AST (U/L)', 'FGF21 ', 'PTN', 'SEX']].copy()
X['intercept'] = 1

# Compute VIF for each column (excluding intercept if desired)
vif_data = pd.DataFrame({
    'variable': X.columns,
    'VIF': [variance_inflation_factor(X.values, i) for i in range(X.shape[1])]
})

# Show VIF table
print(vif_data)
```

***# Statistics metrics for the logit model for MASH discrimination and internal validation through bootstrapping using Python 3.12***

```
# 1. Load and prepare data
df = pd.read_excel('Corr&OlinkNASH_34_markers_all.xlsx')
cols = ['AST (U/L)', 'FGF21 ', 'PTN', 'SEX', 'diagnosis_outcome']
df = df.dropna(subset=cols).copy()

# 2. Compute linear predictor and predicted probability
df['XB'] = (7.57695248874183
            + 0.066647649512763 * df['AST (U/L)']
            + 0.614305598172392 * df['FGF21 ']
            + 0.465190705138323 * df['PTN']
            + 1.66004937491936 * df['SEX'])
df['p_hat'] = 1 / (1 + np.exp(-df['XB']))

y_true = df['diagnosis_outcome'].values
y_score = df['p_hat'].values

# 3. Nominal metrics: AUC, sensitivity, specificity, accuracy
fpr_nom, tpr_nom, thr_nom = roc_curve(y_true, y_score)
auc_nom = roc_auc_score(y_true, y_score)
youden = tpr_nom - fpr_nom
opt_idx = np.argmax(youden)
thresh_opt = thr_nom[opt_idx]
```

```
y_pred = (y_score >= thresh_opt).astype(int)
tn, fp, fn, tp = confusion_matrix(y_true, y_pred).ravel()
sens_nom = tp / (tp + fn)
spec_nom = tn / (tn + fp)
acc_nom = accuracy_score(y_true, y_pred)

# 4. Bootstrap internal validation: metrics and ROC curves
n_boot = 1000
rng = np.random.RandomState(42)
boot_aucs, boot_sens, boot_spec, boot_acc = [], [], [], []
all_tprs = []
mean_fpr = np.linspace(0, 1, 100)

for i in range(n_boot):
    idx = rng.randint(0, len(y_true), len(y_true))
    train = df.iloc[idx]
    test = df.drop(train.index, errors='ignore')
    if len(test)==0 or len(np.unique(test['diagnosis_outcome']))<2:
        continue
    model = LogisticRegression(C=1e12, solver='lbfgs', max_iter=1000)
    X_tr = train[['AST (U/L)', 'FGF21 ', 'PTN', 'SEX']]
    y_tr = train['diagnosis_outcome']
    model.fit(X_tr, y_tr)
    X_te = test[['AST (U/L)', 'FGF21 ', 'PTN', 'SEX']]
    y_te = test['diagnosis_outcome']
    p_te = model.predict_proba(X_te)[:,1]
    fpr_bs, tpr_bs, _ = roc_curve(y_te, p_te)
    boot_aucs.append(roc_auc_score(y_te, p_te))
    tpr_interp = np.interp(mean_fpr, fpr_bs, tpr_bs)
    tpr_interp[0] = 0.0
    all_tprs.append(tpr_interp)
    youden_bs = tpr_bs - fpr_bs
    thr_idx = np.argmax(youden_bs)
    thr_bs = _[thr_idx]
    y_pred_bs = (p_te >= thr_bs).astype(int)
    tn_bs, fp_bs, fn_bs, tp_bs = confusion_matrix(y_te, y_pred_bs).ravel()
    boot_sens.append(tp_bs / (tp_bs + fn_bs))
    boot_spec.append(tn_bs / (tn_bs + fp_bs))
    boot_acc.append(accuracy_score(y_te, y_pred_bs))

# Compute bootstrap summary metrics
mean_auc_bs = np.mean(boot_aucs)
ci_auc_bs = np.percentile(boot_aucs, [2.5, 97.5])
mean_sens_bs = np.mean(boot_sens)
```

```
ci_sens_bs = np.percentile(boot_sens, [2.5, 97.5])
mean_spec_bs = np.mean(boot_spec)
ci_spec_bs = np.percentile(boot_spec, [2.5, 97.5])
mean_acc_bs = np.mean(boot_acc)
ci_acc_bs = np.percentile(boot_acc, [2.5, 97.5])
mean_tpr = np.mean(all_tprs, axis=0)
std_tpr = np.std(all_tprs, axis=0)

# 5. Styled combined ROC plot
plt.figure(figsize=(8,6))
plt.plot(fpr_nom, tpr_nom, color='#2E86AB', lw=2.5, label=f'Nominal ROC (AUC =
{auc_nom:.3f})')
plt.plot(mean_fpr, mean_tpr, color='#A23B72', lw=2.5, label=f'Bootstrap Mean ROC
(AUC = {mean_auc_bs:.3f})')
plt.fill_between(mean_fpr, mean_tpr-std_tpr, mean_tpr+std_tpr, color='#A23B72',
alpha=0.15)
plt.plot([0,1],[0,1], linestyle='--', color='#6C757D', lw=1.5, alpha=0.7)
plt.xlabel('False Positive Rate', fontsize=12, fontweight='bold')
plt.ylabel('True Positive Rate', fontsize=12, fontweight='bold')
plt.title('ROC Curve: Nominal vs Bootstrap', fontsize=14, fontweight='bold',
pad=15)
plt.legend(loc='lower right', shadow=True)
plt.grid(True, linestyle=':', linewidth=0.7, alpha=0.3)
plt.tight_layout()
plt.savefig('roc_nom_bootstrap.png', dpi=300)
plt.show()

# 6. Print summary metrics
print(f"Nominal AUC: {auc_nom:.3f} (Sens: {sens_nom:.3f}, Spec: {spec_nom:.3f},
Acc: {acc_nom:.3f})")
print(f"Bootstrap AUC: {mean_auc_bs:.3f} CI [{ci_auc_bs[0]:.3f},
{ci_auc_bs[1]:.3f}] ")
print(f"Bootstrap Sens: {mean_sens_bs:.3f} CI [{ci_sens_bs[0]:.3f},
{ci_sens_bs[1]:.3f}]")
print(f"Bootstrap Spec: {mean_spec_bs:.3f} CI [{ci_spec_bs[0]:.3f},
{ci_spec_bs[1]:.3f}]")
print(f"Bootstrap Acc: {mean_acc_bs:.3f} CI [{ci_acc_bs[0]:.3f},
{ci_acc_bs[1]:.3f}]")
```
